# Supplementary material for: A novel 3D bilateral filtering algorithm with noise level estimation assisted by multi-temporal SAR
Source: PLoS One. 2025 Feb 19;20(2):e0315395. doi: 10.1371/journal.pone.0315395 (PMC11838908; doi:10.1371/journal.pone.0315395)
Supplement: Attachment — Submitted filename: Response to Reviewers.docx [file pone.0315395.s001.docx]

Dear Editor and Reviewers:

I hope this message finds you well. I would like to express my sincere gratitude for the reviewers’ valuable comments and suggestions on my manuscript entitled “***A Novel Denoising Method Based on Bilateral Filter with Multitemporal SAR-Assisted*** ” (Manuscript ID: ***PONE-D-24-36350***). The constructive comments you provided are highly valuable and offer significant guidance for our study. We fully agree with your suggestions and have made the necessary revisions to the manuscript. These changes are highlighted in the manuscript titled "Revised Manuscript with Track Changes." (***deletions are highlighted in green, and additions in yellow***). We hope you are satisfied with our revisions, and that the updated manuscript meets the publication requirements. **Below are our responses to the suggestions provided by the editor and reviewers.**

**Academic Editor:**

***(1) Please ensure that your manuscript meets PLOS ONE's style requirements, including those for file naming.***

***Response:*** Thank you for your valuable comments, we have corrected the formatting errors present in the article and now the article is formatted to meet the requirements.

***(2) We note that you have indicated that there are restrictions to data sharing for this study. PLOS only allows data to be available upon request if there are legal or ethical restrictions on sharing data publicly.***

***Response:*** Thank you for your valuable feedback. After careful consideration, we have uploaded the data and experimental results used in this study to *https://figshare.com*. The DOI for the dataset is [*https://doi.org/10.6084/m9.figshare.27693714.v1*](https://doi.org/10.6084/m9.figshare.27693714.v1).

***(3) We note that Figures 1, 3, 4, 5, 7, 9, and 10 in your submission contain [map/satellite] images which may be copyrighted. All PLOS content is published under the Creative Commons Attribution License (CC BY 4.0), which means that the manuscript, images, and Supporting Information files will be freely available online, and any third party is permitted to access, download, copy, distribute, and use these materials in any way, even commercially, with proper attribution. For these reasons, we cannot publish previously copyrighted maps or satellite images created using proprietary data, such as Google software (Google Maps, Street View, and Earth).***

***Response:*** Thank you very much for your suggestion. In response, we have added the data source in the first paragraph of Section 4.4.1 (***lines 417-418***). Additionally, we have specified the data source in the descriptions of Figures 5, 6, 7, 8, and 9.

**Reviewer #1:**

***(1) Explain how the present manuscript differs from and improves upon these established previous works. Include a study with your survey papers and be sure to highlight any knowledge gaps that have come up recently.***

***Response:*** Thank you very much for your thoughtful comments on our manuscript. In response, we have revised the summary paragraph in the Introduction (***lines 112-120***). This revised section begins with a summary of the limitations of existing filtering methods, followed by a detailed explanation of how the proposed algorithm effectively addresses these limitations.

***(2) Check and if in case missing, discuss some major sections in the article like the real time/ practical application of the paper, future perspectives, major contribution in the paper, significance of this study.***

***Response:*** Thank you very much for your valuable feedback, which has been instrumental in improving this manuscript. In response to your comments, we have conducted a denoising experiment on real SAR images and compared our algorithm with other commonly used SAR denoising algorithms. The results demonstrate that our algorithm achieves excellent filtering performance, confirming its practical utility (***lines 415-531***).

Additionally, we have rewritten the future perspectives section, where we re-evaluate the limitations of our proposed algorithm and suggest potential solutions (***lines 745-750***). To further highlight the contributions and significance of our algorithm, we have revised the Conclusion section. Here, we briefly outline the original motivation and the issues addressed by our algorithm, summarizing all relevant performance metrics to affirm the algorithm's superiority (***lines 727-744***).

***(3) In the introduction section, include some other research applications as literature.***

***Response:*** We sincerely appreciate the recommended references for this manuscript. After a thorough review, we have incorporated four of the suggested papers into the Introduction as References 7, 8, 29, and 30 (***lines 54, 61, 99, and 101***). These references have greatly enriched the content of our paper:

a) [*https://ieeexplore.ieee.org/abstract/document/7748978*](https://ieeexplore.ieee.org/abstract/document/7748978) *;*
b) [*https://link.springer.com/article/10.1007/s11831-021-09548-z*](https://link.springer.com/article/10.1007/s11831-021-09548-z) *;*
c) [*https://ieeexplore.ieee.org/abstract/document/10411937*](https://ieeexplore.ieee.org/abstract/document/10411937) *;*
d) [*https://ieeexplore.ieee.org/abstract/document/10447792*](https://ieeexplore.ieee.org/abstract/document/10447792)*.*

Since the algorithm proposed in this paper is designed for spatial-domain denoising, we found that including the following references, which also focus on spatial-domain approaches, might affect the manuscript’s coherence. Therefore, we have opted not to add these references at this stage: [*https://www.sciencedirect.com/science/article/pii/S2215098617314003*](https://www.sciencedirect.com/science/article/pii/S2215098617314003) *;*

**Reviewer #2:**

***Work done is very good but the time complexity also must be taken into account ,otherwise it time for preprocessing is more than actual method the proposed method cannot be feasible in applications of GEO-spatial techniques.***

***Response:*** Thank you very much for your valuable comments on this manuscript. We agree that evaluating the time complexity of the algorithm would enhance the completeness of the paper. In response, we have added a dedicated section on time complexity (***lines 715-724***). In this section, we clarify that the time complexity of the algorithm is proportional to $O\left(N^{2}\right)$ when the size of the image to be denoised is $N\times N$. Additionally, we have provided statistics on the execution times of each algorithm used in the denoising experiment. The average execution time for each algorithm is illustrated in the figure below:

As shown in the figure, the execution time of the proposed algorithm is relatively long, and it increases with the number of auxiliary images. This results in the algorithm performing less favorably in comparison to others in terms of execution time. To address this, we have identified the time complexity as a limitation of our algorithm in the future perspectives section (***lines 745-750***), and we have proposed optimizing the algorithm as a key area for future improvement.

**Reviewer #3:**

***(1) The manuscript introduces the Multitemporal SAR-Assisted Bilateral Filtering (MSA-BF) algorithm but fails to provide a strong justification for why this approach is superior to other well-established filters for SAR images. While the paper highlights some of the limitations of the bilateral filter, such as its inability to handle multiplicative noise, the discussion lacks depth regarding the limitations of other comparison filters (e.g., NLM, Lee). A more thorough review of the existing methods and clearer arguments for the necessity of the proposed algorithm are needed.***

***Response:*** We sincerely appreciate your detailed suggestions. In response to your comments, we have provided a more detailed discussion of the limitations of Lee and NLM in the Introduction (***lines 65-67, 84-88***). Additionally, in the summary paragraph of the Introduction, we have summarized the shortcomings of existing box filters (***lines 112-120***). The limitations of the current box filters are as follows:

a) *Most filters perform denoising solely based on the information contained in a single image, often leading to the introduction of excessive extraneous data, which results in blurred edge contours*
b) *These filters typically do not account for the inherent noise levels present in the pixels, resulting in a loss of valuable information after denoising.*

Subsequently, we redefined the design philosophy of the proposed algorithm, clearly outlining the reasons why our algorithm outperforms others.

a) *This approach classifies the pixels to be denoised based on their noise content and designs corresponding filtering methods for each type, effectively reducing the loss of original information.*
b) *To fully utilize the useful information in multi-temporal SAR, the paper introduces four components—range-weight, spatial-weight, confidence-weight, and time-weight. These weights ensure that the denoised pixels retain more useful information, effectively reducing the blurring of contours in the filtered image and preserving more image details.*

***(2) The proposed algorithm introduces several new*** ***components, including confidence-weight, time-weight, and pixel similarity evaluation for range-weight computation. However, the theoretical background and rationale behind these components are not sufficiently explained. The manuscript should include detailed descriptions of how these weights are calculated, their mathematical formulations, and the reasoning behind the choice of these specific techniques.***

***Response:*** Thank you for your valuable suggestions. In response to your feedback, we have revised Section 3.3.1 of the manuscript (***line 280-356***). The revision focuses on describing the design concepts and mathematical principles behind range-weight, spatial-weight, confidence-weight, and time-weight. Additionally, we have rewritten the formulas in this section to make the calculation methods for these four components clearer.

***(3) The manuscript classifies pixels into three categories—strong noise, weak noise, and noise-free pixels—based on noise content along the time axis. However, this approach seems overly simplistic, particularly for handling complex SAR images where noise and signal characteristics may vary significantly across both time and spatial dimensions.***

***Response:*** Thank you for your valuable suggestions. We fully agree with your point, and in response, we have made significant improvements to the pixel classification rule (***line 204-275***). The revised classification rule evaluates the noise content of pixels based on the **temporal and spatial stability** of multi-temporal SAR data. The updated process is as follows:

First, we vectorize the multi-temporal SAR data along the time axis to form a spatial vector matrix. We then calculate the coefficient of variation of this matrix as the **Time Stability Coefficient** (TSC) for the pixels to be denoised. This coefficient reflects the temporal stability of the pixel. At the same time, we construct a 2D pixel block centered on the target pixel in the spatial domain. Using the noise model of SAR pixels, we compare the similarity between this block and a noise-free block. The higher the similarity, the smaller **Spatial Stability Coefficient** (SSC) of the pixel, indicating stronger spatial stability. Finally, we evaluate the noise content of the pixels based on both the TSC and SSC, and classify the pixel into one of three categories: high-noise, low-noise, and noise-free.

The improved classification rule takes both temporal and spatial stability into account, allowing for a more accurate assessment of pixel noise content and, as a result, enhancing the precision of pixel classification.

***(4) The experimental section does not provide sufficient details on how the comparisons between the MSA-BF algorithm and other filters were conducted. For example, the manuscript does not specify the exact parameters used for each comparison filter or the specifics of the multi-temporal SAR data sets used. Clear descriptions of the experimental setup, including the choice of dataset and parameter tuning for each filter, are essential for ensuring the reproducibility of the results.***

***Response:*** Thank you for your thoughtful feedback. In response to your suggestion, we have provided a detailed description of the parameter configurations for all algorithms at the beginning of the "Experiment and Results" section (***lines 411-412***). Additionally, we have included comprehensive descriptions of the data used in both the real SAR image denoising experiment and the simulated SAR image denoising experiment (***lines 415-616, 625-713***).

***(5) While the manuscript reports improvements in ENL (Equivalent Number of Looks) and SSI (Structural Similarity Index), there is little discussion of why these metrics were chosen and what they reveal about the algorithm’s performance. Moreover, the results would benefit from additional quantitative metrics such as PSNR (Peak Signal-to-Noise Ratio) and visual comparisons to provide a more comprehensive evaluation of the filtering performance.***

***Response:*** Thank you for your thoughtful suggestions. In response to your feedback, we have briefly explained the reasons for the selection of ENL vs. SSI in our article( ***lines 585-593,601-616***). And we have added two additional evaluation metrics: PSNR (Peak Signal-to-Noise Ratio) and UIQI (Universal Image Quality Index). Since both of these metrics require a noise-free reference image, we have also included a simulated SAR denoising experiment (***lines 686-713***). Using PSNR and UIQI, we evaluated the results of the simulated experiment, and the evaluation showed that our algorithm achieved the best denoising performance in this simulation.

***(6) “Discussion” section should be added in a more highlighting, argumentative way. The author should analysis the reason why the tested results is achieved.***

***Response:*** Thank you for your helpful suggestions. In response to your feedback, we have elaborated on the reasons behind the denoising results of each algorithm in the experiment and results sections of both the real SAR denoising experiment and the simulated SAR denoising experiment (***lines 458-507, 521-531, 637-650***).

***(7) The authors should clearly emphasize the contribution of the study. Please note that the up-to-date of references will contribute to the up-to-date of your manuscript. The studies named- “Artificial intelligence-based robust hybrid algorithm design and implementation for real-time detection of plant diseases in agricultural environments; Detection of solder paste defects with an optimization‐based deep learning model using image processing techniques”- can be used to explain the methodology and highlight the performance in the study or to indicate the contribution in the “Introduction” section.***

***Response:*** Thank you for your valuable comments, we have included a discussion on the application of deep learning in SAR denoising in the Introduction (***lines 96-105***),and have referenced the two articles you recommended within that section (***line 96***).

***(8) The claim that the proposed algorithm improves filtering in homogeneous regions "by nearly threefold" and enhances performance in large complex regions "by approximately 20%" seems exaggerated without adequate support. The manuscript should provide more rigorous statistical evidence and error analysis to substantiate these claims. Otherwise, the paper risks overclaiming the benefits of the proposed approach.***

***Response:*** Thank you for your valuable feedback. Based on your suggestions, we have removed the comparison of metrics from the abstract and rewritten the analysis sections for ENL and SSI (***lines 587-593, 613-616***). In the simulated SAR denoising experiment, we evaluated the performance of each denoising algorithm using PSNR and UIQI and provided an appropriate analysis of the results (***lines 708-713***).

***(9) The manuscript does not provide any analysis of the computational complexity of the MSA-BF algorithm. Given that the algorithm introduces several new components, such as confidence-weight and time-weight, it is important to discuss the computational trade-offs involved in achieving the reported performance improvements. An analysis of runtime or resource consumption compared to other filters would be a valuable addition.***

***Response:*** Thank you very much for your valuable comments on this manuscript. This issue is similar to the comments raised by Reviewer 2. We agree that assessing the time complexity of the algorithm adds to the overall completeness of the paper. In response, we have added a dedicated section on time complexity (***lines 715-724***). In this section, we clarify that the time complexity of the algorithm is proportional to $O\left(N^{2}\right)$ when the size of the image to be denoised is $N\times N$. Additionally, we have provided statistics on the execution times of each algorithm used in the denoising experiment. The average execution time for each algorithm is illustrated in the figure below:

As shown in the figure, the execution time of the proposed algorithm is relatively long, and it increases with the number of auxiliary images. This results in the algorithm performing less favorably in comparison to others in terms of execution time. To address this, we have identified the time complexity as a limitation of our algorithm in the future perspectives section (***lines 745-750***), and we have proposed optimizing the algorithm as a key area for future improvement.

***(10) The results section is not well-structured, making it difficult to follow the comparisons and assess the performance of the MSA-BF algorithm. The paper would benefit from organizing the results into clearer subsections with tables, graphs, and visual comparisons for each Earth surface type. This would improve readability and allow for a more transparent presentation of the findings.***

***Response:*** Thank you for your insightful suggestions. In response, we have highlighted key regions within the denoising results and rewritten the Results section to analyze these outcomes based on the denoising principles of each algorithm. This revision clarifies the strengths and weaknesses of each method (***lines 460-507, 637-650***).

**Reviewer #4:**

***(1) The author did not describe the drawbacks of each conventional technique in the introduction paragraph.***

***Response:*** We sincerely appreciate your detailed suggestions. In response, we have expanded the discussion on the limitations of Lee and NLM in the Introduction (***lines 65-67, 84-88***).

***(2) Add the problem definition of this work in the introduction paragraph because it is not clear.***

***Response:*** Thank you for your valuable feedback. In the summary paragraph of the Introduction, we have summarized the shortcomings of existing box filters (***lines 112-120***). The limitations of the current box filters are as follows:

a) *Most filters perform denoising solely based on the information contained in a single image, often leading to the introduction of excessive extraneous data, which results in blurred edge contours*
b) *These filters typically do not account for the inherent noise levels present in the pixels, resulting in a loss of valuable information after denoising.*

Subsequently, we redefined the design philosophy of the proposed algorithm, clearly outlining the reasons why our algorithm outperforms others.

a) *This approach classifies the pixels to be denoised based on their noise content and designs corresponding filtering methods for each type, effectively reducing the loss of original information.*
b) *To fully utilize the useful information in multi-temporal SAR, the paper introduces four components—range-weight, spatial-weight, confidence-weight, and time-weight. These weights ensure that the denoised pixels retain more useful information, effectively reducing the blurring of contours in the filtered image and preserving more image details.*

***(3) Please include the references for all equations.***

***Response:*** Thank you very much for pointing out the formatting errors in our manuscript. We have now properly cited all formulas, figures, and tables, and have corrected all formatting issues throughout the paper.

***In addition to the corrections mentioned above, we have made the following additional revisions:***

a) *To better highlight the advantages of our proposed algorithm, we have modified the title to "A Novel 3D Bilateral Filtering Algorithm with Noise Level Estimation Assisted by Multi-Temporal SAR."*
b) *To clarify the focus of this study, we have rewritten the abstract* (***lines 27-46***).
c) *We have adjusted the numbering of the figures, formulas, tables, and references in the manuscript.*

I believe these revisions have significantly improved the manuscript, and I hope the revised version meets your expectations. I have attached the updated manuscript along with a marked-up version that highlights the changes made.

Thank you for considering the revised manuscript for publication in ***PLOS ONE***. I look forward to hearing from you.

Best regards,
Authors
